# Supplementary material for: Content-rich biological network constructed by mining PubMed abstracts
Source: BMC Bioinformatics. 2004 Oct 8;5:147. doi: 10.1186/1471-2105-5-147 (PMC528731; doi:10.1186/1471-2105-5-147)
Supplement: Additional File 2 — The original results of the above study (non-essential files are deleted to keep the file size under the limit set by BMC bioinformatics). [file 1471-2105-5-147-S2.bz2 › chilibotAdditionalFile2/dip05/454ID10477718E10466/html/CCL11_CCR5.html]

 


 **CCL11** and **CCR5** 
  
Found 50 abstracts in PubMed, retrieved 05.  
 

 What does Google say? 
 PDF only 
| .edu only 

---

- Am J Pathol, 2003   **Chemokine Production and Leukocyte Recruitment to the Lungs of Paracoccidioides brasiliensis Infected Mice Is Modulated by Interferon gamma.** .
  Chemokines and chemokine receptors play a role in cell recruitment during granulomatous inflammatory reactions.
  Here, we evaluated the expression of chemokines and chemokine receptors and their regulation by IFN gamma in the course of Paracoccidioides brasiliensis Pb infection in mice.
  We found an association between KC and MIP 1alpha CCL3 production and neutrophil infiltration in the lungs of Pb infected mice during the early acute phase of infection.
  High levels of RANTES CCL5, MCP 1 CCL2, IP 10 CXCL10, and Mig CXCL9 simultaneously with mononuclear cell infiltration in the lungs was found.
  In the absence of IFN gamma GKO mice we observed increased production of KC and MIP 1alpha and chronic neutrophilia.
  Moreover, we found a change in the chemokine receptor profiles expressed by wild type WT versus GKO animals.
  Increased expression of CXCR3 and **CCR5**, and low levels of CCR3 and CCR4 were observed in the lungs of Pb infected WT mice.
  . . . the opposite effect was observed in the lungs of GKO mice.
  Consistent with these results, infected cells from WT mice preferentially migrated in response to IP 10 CXCR3 ligand , while those from GKO mice migrated in response to eotaxin **[CCL11]** **CCL11** CCR3 ligand .
  These results suggest that IFN gamma modulates the expression of chemokines and chemokine receptors as well as the kind of cells that infiltrate the lungs of Pb infected mice.

  - Biochem Pharmacol, 2003   **Expression and characterization of the chemokine receptor CCR2B from rhesus monkey.** .
    Species selectivity of chemokine receptor antagonists is a potential deterrent to making preclinical assessments in vivo.
    To determine if rhesus monkey disease models could support these assessments, we pharmacologically and functionally characterized recombinant rhesus CCR2B receptor.
    For these studies we obtained the CCR2B coding region by PCR from genomic rhesus DNA and expressed the receptor as stable transfectants in Chinese Hamster Ovary cells.
    The surface expression of recombinant rhesus CCR2B was detected by flow cytometry using a commercially available monoclonal anti hCCR2B antibody.
    This antibody was used to detect rhCCR2B on monocytes in peripheral blood mononuclear cell preparations from rhesus whole blood.
    The recombinantly expressed CCR2B exhibited similar high affinity binding to the CCR2 chemokine ligands from rhesus and human 125I rhMCP 1 K d =433 14pM and 125I hMCP 1 K d =550 256pM .
    In competition binding, the receptor exhibited selective high affinity binding to the monocyte chemoattractant protein MCP family chemokines with little affinity for most other members of the CC family of chemokines.
    One exception was eotaxin **[CCL11]**, a high affinity ligand for CCR3, which bound to rhesus CCR2B receptor K i =1467 205pM .
    Chemokines which exhibited binding affinity for the receptor were tested for their ability to induce intracellular calcium release.
    In these experiments the relative potencies of the MCP family of chemokines for rhCCR2B were similar to the observed binding affinities.
    In contrast, eotaxin **[CCL11]** was functionally inactive as an antagonist or agonist to this receptor.
    TAK 799 N,N dimethyl N 4 2 4 methylphenyl dihydro 5H benzocyclohepten 8 yl carbonyl amino benzyl tetrahydro 2H pyran 4 aminium chloride , a dual CCR2 **CCR5** antagonist, demonstrated high affinity for the rhesus CCR2B in competition with 125I hMCP 1 binding to the receptor K i =0.5nM and also potently blocked the MCP 1 induced calcium mobilization mediated through the receptor.

    - Verh K Acad Geneeskd Belg, 2002    **Role of chemokines in the HIV infection process** .
      In order to infect a target cell, the HIV envelope glycoprotein gp 120 has to interact with both the cellular receptor CD4 and an HIV coreceptor,i.e. the CC or CXC chemokine receptor **CCR5** or CXCR4.
      Both coreceptors were immediately recognized as novel targets for anti HIV therapy.
      Blocking these coreceptors would protect the cell from viral entry and would reduce the viral transmission and pathogenesis.
      Here we describe the purification and characterization of natural chemokine variants and compare their antiviral activity.
      In addition, the role of proteases for the processing of the CC chemokines RANTES, eotaxin **[CCL11]**, MDC and MIP 1 alpha and of the CXC chemokine SDF 1 are studied.
      The MIP 1 alpha isoform LD78 beta, that was purified form natural sources, inhibited HIV infection completely in **CCR5** transfected cells, mononuclear leukocytes and purified monocytes at low ng ml concentrations.
      This research will make it feasible to develop specific chemokine analogs that block HIV entry.
      Deciphering the processes that play a role during the complicated interactions between HIV gp120 and the cellular membrane may lead to a more efficient treatment of HIV infections.

      - Eur J Immunol, 2003   We have previously shown that the CC chemokine 1 309 protects mouse thymic lymphomas against corticoid induced apoptosis.
        Here, we analyzed the signal transduction pathways involved in this activity on BW5147 lymphoma.
        Inhibition of the CCL1 **[CCL11]** activity by pertussis toxin suggested the involvement of a G protein coupled chemokine receptor.
        The role of CCRS was supported by the observation that vMIP I, another CCR8 ligand identified from the genome of a T cell transforming herpes virus, shared CCL1 **[CCL11]** anti apoptotic activity.
        In addition to CCR8, BW5147 cells also expressed the CXCR4 receptor but its ligand, SDF 1 showed only a modest anti apoptotic activity.
        Other chemokines acting on CCR2, CCR4 and **CCR5** failed to protect against apoptosis and to induce BW5147 chemotaxis, suggesting that these receptors were not functionally expressed.
        By contrast, both CCL1 **[CCL11]** and vMIP I up regulated ERK1 2 MAPK phosphorylation in BW5147 cells.
        Further analysis demonstrated that CCL1 **[CCL11]** activates the MAPK pathway in CCR8 transfected CHO cells.
        The implication of this pathway was confirmed by the fact that PD98059, an inhibitor of MEK kinases, as well as a dominant negative isoform of the M RAS protein specifically blocked the anti apoptotic activity of CCL1 **[CCL11]**.

        - Xenotransplantation, 2003   **The role of chemokines and their receptors in the rejection of pig islet tissue xenografts.** .
          Solomon MF, Kuziel WA, Mann DA, Simeonovic CJ.
          The role of chemokines and their receptors in the rejection of pig islet tissue xenografts.
          Xenotransplantation 2003.
          10 164 177.
          Copyright Blackwell Munksgaard, 2003 The mechanism by which inflammatory cells are recruited to pig islet tissue proislet xenografts was investigated by examining the intragraft mRNA expression of murine alpha and beta chemokines in CBA H mice from days 3 to 10 post transplant.
          Xenograft rejection was associated with early intragraft transcript expression for monocyte chemotactic protein 1 MCP 1 3 to 5 days , IP 10 3 to 4 days and macrophage inflammatory protein 1alpha MIP 1alpha 3 to 5 days and subsequent expression of eotaxin **[CCL11]** days 4 to 10 , MIP 1beta days 4 and 5 and regulated on activation, normal T cell expressed and secreted RANTES days 4 to 6 mRNA.
          This pattern was consistent with the early recruitment of macrophages MCP 1, MIP 1alpha , the influx of CD4 T cells MCP 1, MIP 1alpha, MIP 1beta, IP 10 and RANTES and the characteristic infiltrate of eosinophils eotaxin **[CCL11]** and RANTES associated with islet xenograft rejection.
          Inhibition of beta chemokine signaling in CCR2 mice which lack the major co receptor for MCP 1 resulted in retarded macrophage and CD4 T cell recruitment, enhanced eosinophil influx and a minor delay in rejection, compared with wildtype mice.
          there was little effect on leukocyte infiltration in xenografts harvested from **CCR5** mice lacking the co receptor for MIP 1alpha, MIP 1beta and RANTES .
          The impeded migration of leukocytes into xenografts in CCR2 hosts was associated with delayed intragraft expression of MCP 1 and RANTES mRNA.
          absence of MCP 1 CCR2 mediated signaling led to enhanced intragraft expression of MCP 1, MIP 1alpha and MIP 1beta mRNA.
          These findings suggest that MCP 1 plays an important role in regulating macrophage and CD4 T cell infiltration to xenograft sites via the CCR2 signaling pathway.
          Additional treatment of xenografted CCR2 transplant recipients with anti interleukin IL 4 and anti IL 5 mAbs further delayed xenograft rejection demonstrating the potential for combined antirejection strategies in facilitating pig islet xenotransplantation.
